# Accidental Discovery of Ocular Cicatricial Pemphigoid

**DOI:** 10.7759/cureus.77425

**Published:** 2025-01-14

**Authors:** Amine Razzak, Hala Ait Ammar, Mohamed Bouazza, Mohamed Elbelhadji

**Affiliations:** 1 Department of Ophthalmology, Mohammed VI University of Sciences and Health, Casablanca, MAR; 2 Research Unit, Mohammed VI Center for Research and Innovation, Rabat, MAR

**Keywords:** ankyloblepharon, eyelid malposition, ocular cicatricial pemphigoid, ocular surface, symblepharon

## Abstract

Ocular cicatricial pemphigoid (OCP) is a chronic systemic autoimmune dermatosis characterized by progressive and fibrosing inflammation of the conjunctiva. We report the case of a 54-year-old female patient who presented with bilateral and progressive visual acuity loss. Clinical examination revealed trichiasis, bilateral symblepharon, bilateral superficial punctate keratitis, corneal opacity, and bilateral cataracts. Systemic evaluation identified erosive gingivitis and scarred bullous lesions. Histopathological and immunofluorescence examinations confirmed the diagnosis of OCP. The patient was treated with oral corticosteroids, bolus cyclophosphamide, dexamethasone eye drops, and moisturizing agents. Symblepharon surgery and photoablation of the trichiasic eyelashes were performed after three months. At 12 months, no recurrence was observed, and cataract surgery was proposed following a 6-month remission period. OCP is a systemic, progressive, and potentially blinding condition requiring early and multidisciplinary management to control inflammation and improve outcomes. However, it’s frequently diagnosed late, especially in the absence of significant visual acuity loss, highlighting the need for increased clinical vigilance.

## Introduction

Ocular cicatricial pemphigoid (OCP) is a chronic, progressive autoimmune dermatological condition characterized by fibrosing inflammatory damage to the conjunctiva [1]. This rare systemic disorder can result in blindness due to severe corneal complications. Extra-ocular involvement, including the skin and mucous membranes, is often observed and may become life-threatening, particularly in cases involving the larynx or esophagus [2].

The diagnosis of OCP relies on clinical signs affecting ocular and extra-ocular sites, confirmed by histopathological studies. However, even in cases with conjunctival involvement, diagnosis can be challenging due to non-specific clinical manifestations [3]. Early initiation of treatment is crucial and typically requires a multidisciplinary approach, frequently involving systemic immunosuppressants. Despite rigorous monitoring, clinical outcomes are often unsatisfactory [4].

We present the case of a patient in whom OCP was fortuitously discovered during a preoperative evaluation for cataract surgery.

## Case presentation

We present the case of a 54-year-old woman with no history of physical or chemical trauma or drug use, who consulted for progressive bilateral visual loss. Ophthalmologic examination revealed visual acuity of 2/10 in the right eye and 1/10 in the left. Ocular motility was reduced bilaterally, with more significant restriction in the left eye. Examination of the eyelids revealed trichiasis and bilateral symblepharon, occupying the inferior conjunctival fornix. These findings were classified as stage IIB in the right eye and IIIB in the left, according to Tauber and Foster's classification (Table 1).

**Table 1:** Severity score of conjunctival fibrosis according to the Tauber and Foster classification Adapted from: [5]

Stage		Clinical manifestations
I		Superior tarsal fibrosis
II	A	Filling 0 to 25% of the depth of the inferior conjunctival fornix
	B	Filling 25 to 50% of the depth of the inferior conjunctival fornix
	C	Filling 50 to 75% of the depth of the inferior conjunctival fornix
	D	Filling 75 to 100% of the depth of the inferior conjunctival fornix
III	A	Symblepharon covering 0 to 25% of the length of the conjunctival fornix
	B	Symblepharon covering 25 to 50% of the length of the conjunctival fornix
	C	Symblepharon covering 50 to 75% of the length of the conjunctival fornix
	D	Symblepharon covering 75 to 100% of the length of the conjunctival fornix
IV		Ankyloblepharon

**Figure 1:**
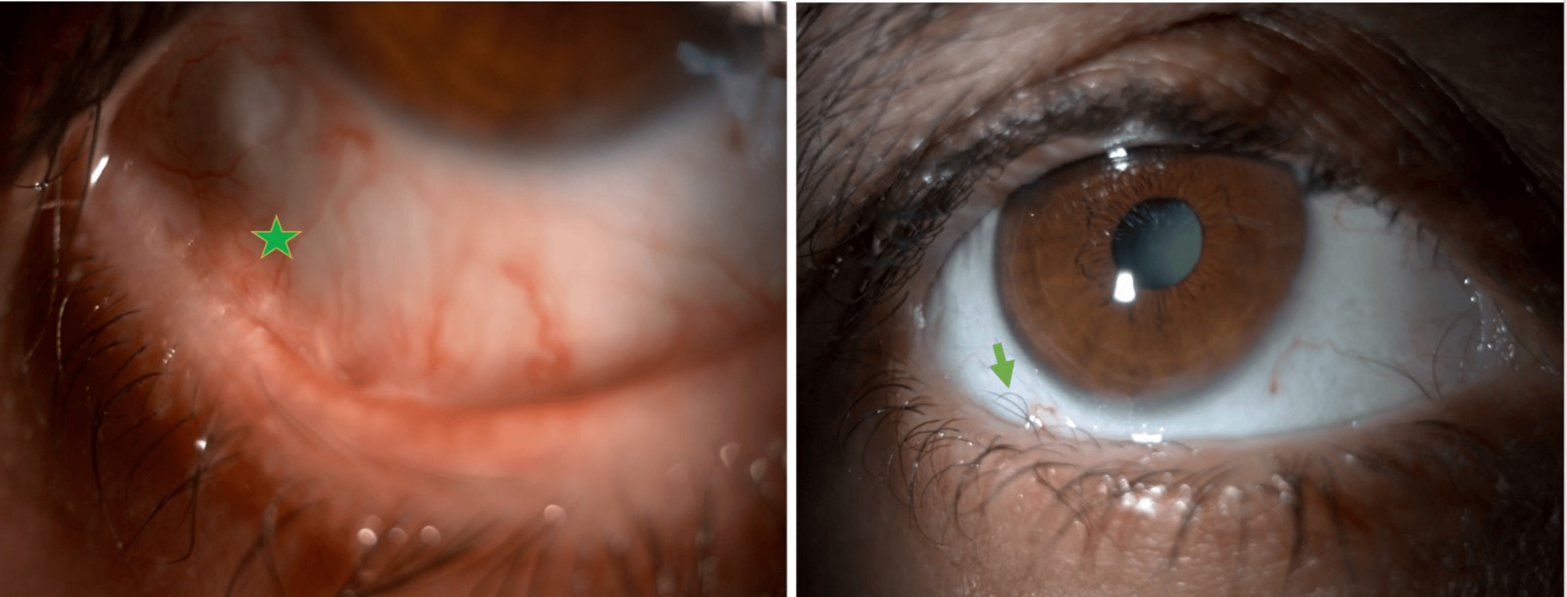
Preoperative appearance of the right eye Symblepharon (asterisk) and trichiasis (arrow)

Corneal examination revealed bilateral superficial punctate keratitis and a paracentral corneal opacity in the left eye that spared the visual axis (Figures 1a, 1b). Additionally, bilateral nuclear cataracts were noted, accounting for the decrease in visual acuity.

Systemic examination revealed erosive gingivitis and bullous, scarring lesions on the trunk. Histopathological examination of the skin lesions demonstrated subepidermal cleavage, and immunofluorescence revealed linear deposits of C3 and IgG at the dermo-epidermal junction, confirming a diagnosis of OCP.

**Figure 2:**
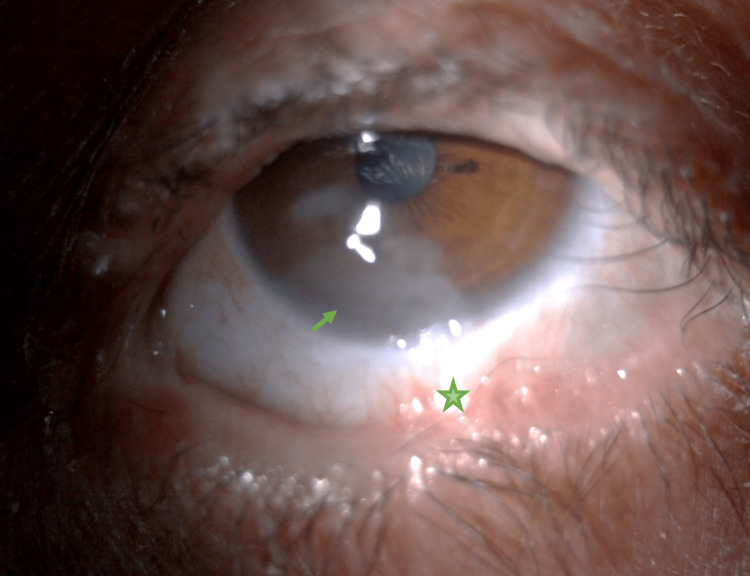
Preoperative appearance of the left eye Filling of the conjunctival fornix (asterisk) and corneal opacity (arrow)

Given the severity of the bilateral ocular involvement and the systemic nature of the disease, the patient was initiated on oral prednisolone at a dose of 1 mg/kg/day, followed by a gradual tapering regimen. To further control inflammation and prepare the ocular surface for cataract surgery, three bolus doses of cyclophosphamide (Endoxan®) were administered. Locally, dexamethasone eye drops were prescribed with a tapering regimen, alongside lubricating eye drops for symptomatic treatment. Figure 2 shows the preoperative appearance of the left eye.

**Figure 3:**
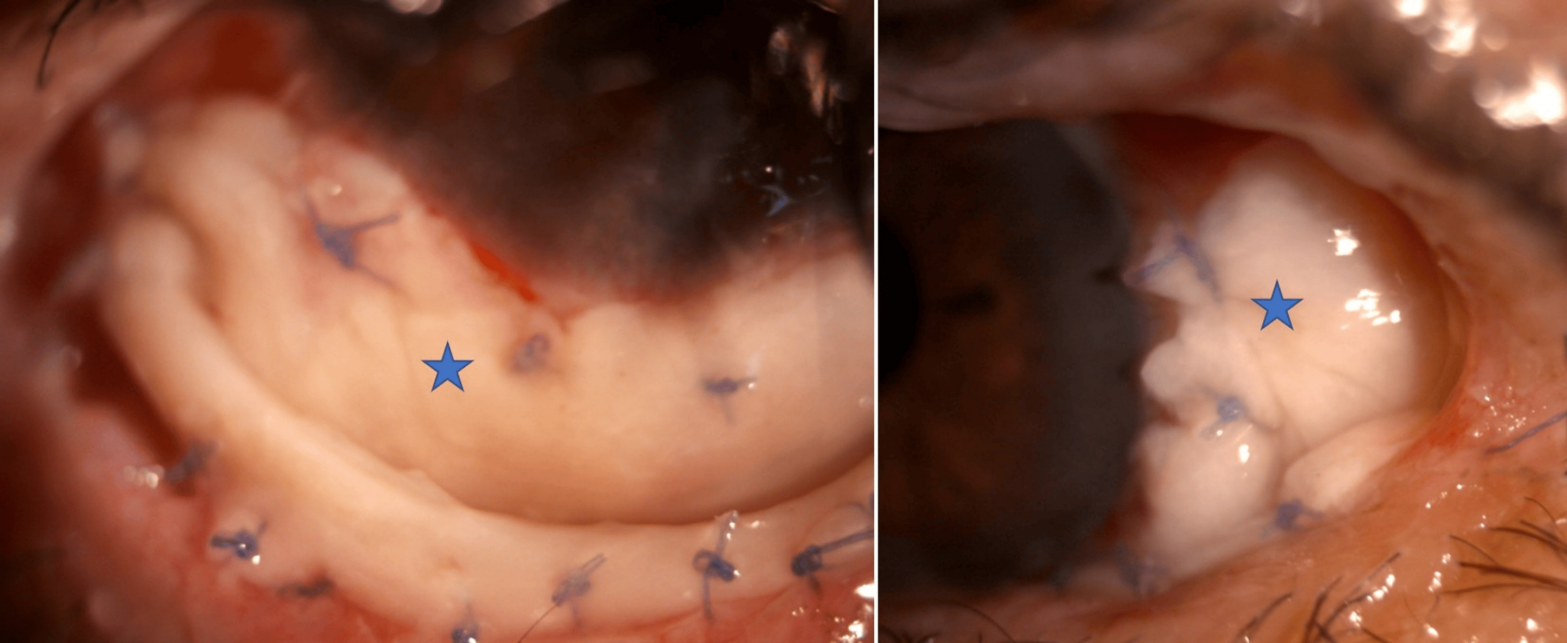
Immediate postoperative appearance of the left eye Labial mucosa graft (asterisk)

After three months, surgical intervention for the symblepharon in the left eye was performed. This involved releasing adhesions and reconstructing the inferior and temporal conjunctival fornices using an autograft of labial mucosa and placing a symblepharon ring. Postoperative outcomes were favorable, with good vascularization of the graft and no signs of exacerbated inflammation (Figure 3).

The patient also underwent photoablation of the trichiasis in both eyes. Over a 12-month follow-up, there was no recurrence of fibrosing conjunctivitis. Cataract surgery for both eyes was proposed after achieving six months of disease remission.

## Discussion

Ocular cicatricial pemphigoid is a rare disease primarily affecting the elderly, with a female predominance. It is part of a group of chronic inflammatory diseases of the skin and mucous membranes, commonly referred to as mucous membrane pemphigoid (MMP). This autoimmune disease is characterized by the formation of subepithelial blisters that progressively lead to fibrotic scarring of the conjunctiva. Clinical manifestations include xerosis, symblepharon, and eyelid malposition, which can result in irreversible corneal opacification [6].

The diagnosis is often suspected based on non-specific ocular and extra-ocular clinical signs. Ocular surface involvement is bilateral and asymmetrical and can lead to corneal opacification and blindness [7]. The disease is marked by chronic, progressive, fibrosing conjunctival inflammation which, if untreated, can cause fornix shrinkage, symblepharon, or even ankyloblepharon [8].

Extra-ocular involvement occurs in approximately half of the cases and may affect the skin, oral, esophageal, nasal, laryngeal, anal, and genital mucous membranes. These manifestations can lead to symptoms such as dysphagia, digestive issues, and dyspareunia [3]. The severity of extra-ocular involvement varies depending on the extent and location of the inflammation, and in some cases, the disease can be life-threatening. This highlights the importance of close monitoring and multidisciplinary management.

The insidious nature and non-specific clinical signs of OCP often delay diagnosis [9]. As a result, the disease may remain asymptomatic for an extended period, even with conjunctival involvement. In our case, the patient's consultation was prompted by decreased visual acuity due to cataracts. Histopathological examination of the conjunctiva or skin lesions is essential for diagnosis, particularly in the absence of pathognomonic signs. Direct immunofluorescence remains the gold standard for detecting specific linear anti-basement membrane antibodies [10].

Treatment for OCP requires a multidisciplinary approach aimed at controlling conjunctival inflammation and managing complications. Ocular treatment strategies include lubricating agents, topical corticosteroids, and therapeutic soft contact lenses to reduce inflammatory episodes, though no local treatment has been proven to halt disease progression [3].

Systemic corticosteroids help manage inflammation and prevent fibrosis, but long-term monotherapy carries significant risks of systemic complications. Immunosuppressants such as cyclophosphamide or methotrexate are commonly combined with corticosteroids to mitigate side effects. Monoclonal antibodies like rituximab have demonstrated efficacy in stabilizing refractory cases, although their high cost limits widespread use [4].

Surgical intervention is often required to address complications arising from conjunctival fibrosis, eyelid malposition, or corneal opacification. However, inflammation must be adequately controlled before surgery to prevent postoperative inflammatory flare-ups [10]. Symblepharon management typically involves adhesion release and ocular surface reconstruction using buccal or labial mucosa grafts. Amniotic membrane transplantation has been proposed for ocular surface reconstruction though its use is limited by the risk of exacerbating postoperative inflammation [11].

In advanced cases, osteo-odonto-keratoprosthesis has been proposed as a last resort to maintain functional vision. Falcinelli et al. reported an anatomical success rate of up to 85% for this technique in severe corneal blindness [12]. However, functional outcomes remain suboptimal due to progressive visual degradation. Consequently, this high-risk procedure is reserved for cases where corneal transplantation is contraindicated.

For patients with associated cataracts, surgery should be delayed until inflammation is well-controlled to minimize postoperative complications. In the case we present, despite the relatively late and incidental discovery of the disease, a combination of medical and surgical management successfully controlled inflammation, treated complications, and halted disease progression, significantly improving the patient's quality of life.

This case underscores the importance of considering OCP in the differential diagnosis of chronic conjunctival inflammation, particularly in elderly patients. It also highlights the need for heightened awareness among clinicians and the value of multidisciplinary collaboration in managing such complex conditions. Early recognition and intervention are crucial for preventing severe complications, preserving vision, and enhancing overall quality of life.

## Conclusions

Ocular cicatricial pemphigoid is a rare, potentially blinding autoimmune disease with significant physical and psychological impact. Its rarity and variable clinical presentation make diagnosis challenging, particularly in our context, where it is often identified late in the disease course, especially in the absence of significant visual impairment.

Early and multidisciplinary therapeutic management is crucial for promptly controlling inflammation. This approach requires a combination of medical therapies, surgical interventions, and consistent follow-up. Although advancements in immunosuppressive treatments have improved prognosis, long-term outcomes remain unpredictable, highlighting the importance of ongoing vigilance and regular monitoring. Further research is essential to deepen our understanding of the pathogenesis of OCP and to develop more effective therapeutic strategies aimed at improving patient outcomes and long-term prognoses.
